# Estimating Mental Health Conditions of Patients with Opioid Use Disorder

**DOI:** 10.1155/2019/8586153

**Published:** 2019-09-26

**Authors:** Christopher Minnerly, Steven L. Bressler, Ibrahim M. Shokry, Rui Tao

**Affiliations:** ^1^Charles E. Schmidt College of Medicine, Florida Atlantic University, Boca Raton, FL, USA; ^2^FHE Health, Deerfield Beach, FL, USA; ^3^Center for Complex Systems and Brain Sciences, Florida Atlantic University, Boca Raton, FL, USA; ^4^Department of Psychology, Florida Atlantic University, Boca Raton, FL, USA; ^5^Ross University School of Veterinary Medicine, Basseterre, Saint Kitts and Nevis

## Abstract

**Objectives:**

Noninvasive estimation of cortical activity aberrance may be a challenge but gives valuable clues of mental health in patients. The goal of the present study was to characterize specificity of electroencephalogram (EEG) electrodes used to assess spectral powers associated with mental health conditions of patients with opioid use disorder.

**Methods:**

This retrospective study included 16 patients who had been diagnosed with opioid use disorder in comparison with 16 sex- and age-matched healthy controls. EEG electrodes were placed in the frontal (FP1, FP2, F3, F4, F7, F8, and Fz), central (C3, C4, and Cz), temporal (T3, T4, T5, and T6), parietal (P3, P4, and Pz), and occipital scalp (O1 and O2). Spectral powers of *δ*, *θ*, *α*, *β*, and γ oscillations were determined, and their distribution was topographically mapped with those electrodes on the scalp.

**Results:**

Compared to healthy controls, the spectral powers at low frequencies (<8 Hz; *δ* and *θ*) were increased in most electrodes across the scalp, while powers at the high frequencies (>12 Hz; *β* and γ) were selectively increased only at electrodes located in the frontal and central scalp. Among 19 electrodes, F3, F4, Fz, and Cz were highly specific in detecting increases in *δ*, *θ*, *β*, and γ powers of patients with opioid use disorders.

**Conclusion:**

Results of the present study demonstrate that spectral powers are topographically distributed across the scalp, which can be quantitatively characterized. Electrodes located at F3, F4, Fz, and Cz could be specifically utilized to assess mental health in patients with opioid use disorders. Mechanisms responsible for neuroplasticity involving cortical pyramidal neurons and *μ*-opioid receptor regulations are discussed within the context of changes in EEG microstates.

## 1. Introduction

The opioid epidemic in the US has hit an all-time high in recent years, with rates of affliction exponentially increasing. In 2016, an estimated 12 million people have used opioids for a variety of purposes, and approximately 2.1 million of those individuals suffer from mental disorders due to opioid uses [1]. The rates of opioid use disorder are projected to become worse in the next few years [2].

Opioid use disorder is often diagnosed with psychiatric evaluation based on the fifth edition of the Diagnostic and Statistical Manual of Mental Disorders (DMS-5; [3]). How brain activity is changed due to opioid use can only be determined with brain imaging techniques such as positron emission tomography (PET; [4]). However, high operating cost restricts the use of PET scans for the clinical evaluation. Electroencephalogram (EEG), which is commonly used to estimate neuronal disorders in patients with epilepsy or schizophrenia [5, 6], could be a more affordable alternative in estimating brain activity aberrance. EEG signals often reflect spatial and temporal activities of underlying cortical microcircuits consisting of pyramidal glutamatergic neurons, GABAergic interneurons, and subcortical inputs ([7]; also see a review by Cohen [8]). Opioids such as morphine, oxycodone, and heroin, exert their neurological effects mainly through activating *μ*-opioid receptors that reside almost exclusively on GABAergic neurons [9, 10]. The *μ*-opioid receptors are functionally coupled with G protein-gated inwardly rectifying K^+^ (GIRK) channels, and activation of which results in hyperpolarization of GABAergic neurons and decreases in amplitude of inhibitory postsynaptic potentials (IPSPs) [11, 12]. Thus, opioids are considered as central nervous system (CNS) depressants, along with alcohol and benzodiazepines in substance abuse. EEG activity is increased when GABAergic neurons in the cortical microcircuits are disinhibited with opioids [13]. These microcircuits also receive afferent innervations from neurons of deep brain nuclei, which are regulated by opioids on GABAergic neurons [14]. Therefore, in general, there is a reduction in EEG activity following acute opioid administration as being demonstrated with drug-naïve humans [15, 16] and rodents as well [17]. However, long-term opioid administration causes an impairment of GIRK channels and GABAergic neurons [18, 19]. Furthermore, GABAergic hyperfunction is associated with drug dependence [20]. As a result, opioid use disordered individuals are more likely to have mental health conditions [21].

EEG is the summation of electrical signals that are measured as *δ*, *θ*, *α*, *β,* and γ oscillations [22]. This may raise the question as to what oscillations or rhythms are altered in patients with opioid use disorders. At which scalp electrodes are those changes being detected? Currently, is no consensus upon clinical relationship of EEG electrodes and rhythms altered in opioid patients (see reviews [23, 24]). For instance, Wang et al. reported equal increases of *δ*, *θ*, *α*, and *β* powers in almost all EEG electrodes placed on the scalp [25]. Motlagh showed that *β* but not *δ*, *θ*, or *α* oscillations were increased in opioid patients [26]. Although causes of inconsistency between reports are unknown, it suggests that it is necessary and also needed to re-evaluate the relationship of EEG electrodes and rhythms altered in opioid patients. Most recently, EEG analytical methods to investigate effects of drug abuse on *δ*, *θ*, *α*, *β,* and γ oscillations have been successfully developed in our laboratory [27, 28], which make it possible for us to reliably analyze rhythmic data extracted from patients.

We hypothesize that changes in EEG rhythms are characteristic of patients with opioid use disorder; furthermore, such changes occur at specific electrodes, which can be used to assess mental health conditions. Therefore, the aim of the present study was to characterize spectral rhythms recorded at EEG electrodes of patients with opioid use disorder compared to healthy controls. To fulfill this goal, we were first to reveal the normalized distribution of *δ*, *θ*, *α*, *β,* and γ rhythm powers using data from healthy controls. The subsequent analysis was to identify individual electrodes which showed significant changes in *δ*, *θ*, *α*, *β,* or γ powers of patients with opioid use disorders compared to those in healthy controls. Lastly, we examined whether spectral power distribution across the scalp was altered in patients with opioid use disorder.

## 2. Materials and Methods

### 2.1. Study Design

Data were obtained from an electronic medical database at a substance abuse treatment facility (FHE Health, Deerfield Beach, FL, USA), which had gathered ∼1000 cases of information about patients' drug use history, DMS-5 diagnosis, and drug intoxication treatment. In addition, there were 20 cases obtained from healthy subjects with records indicating no substance abuse history. EEG data can be tracked electronically, along with information about detox-related symptoms. Searching with opioid-related keywords (i.e., morphine, heroin, fentanyl, methadone, or oxycodone), we found 350 patients who had records of opioid use history. However, a majority of patients were polysubstance users. After exclusion of alcohol, cocaine, and methamphetamine, only 100 subjects were considered as opioid users. Those with neurodegenerative diseases (e.g., Parkinson's and Alzheimer's), incomplete medical records, or low quality of EEG data were also excluded from the study. To this end, eleven men and five women identified with opioid use disorder were compared with 16 sex- and age-matched healthy controls. As shown in Table 1, patients had at least a three-year history of opioid abuse (average of 7.8 years). As a standard detox procedure, detoxification medications (buprenorphine) at a dosage of 8 mg were given at the time of EEG recordings while abstinent from other opioid use for no more than one week (average of 2.4 days). No medication was given to sixteen healthy controls at EEG recording. The protocols of retrospective analysis of living subjects were approved by the Institutional Review Board (IRB) from Florida Atlantic University (Boca Raton, FL, USA) and Ross University School of Veterinary Medicine.

### 2.2. EEG Data Acquisition

In patients' medical records, EEG recording was performed between 12:00 PM and 4:00 PM. Following instrumental calibration, a case (patient or healthy control) was seated in a comfortable chair in a dimmed recording room, and the EEG procedures were orally instructed. A cap with 19 electrodes (Electro-Cap International, Eaton, OH, USA) was placed on the scalp. To reduce muscle artifacts in the EEG signal, the subject was instructed to assume a comfortable position and to avoid movement. Signals were collected with the band-pass filter of 1–100 Hz at a rate of 256 Hz and amplified with Neurofield's Q20 amplifier (NeuroField Inc., Bishop, CA, USA) using NeuroGuide software (Applied Neuroscience Inc., Tampa, FL, USA). Each subject underwent 10 minutes of EEG recording with eyes closed.

### 2.3. EEG Data Analysis

**Figure 1:**
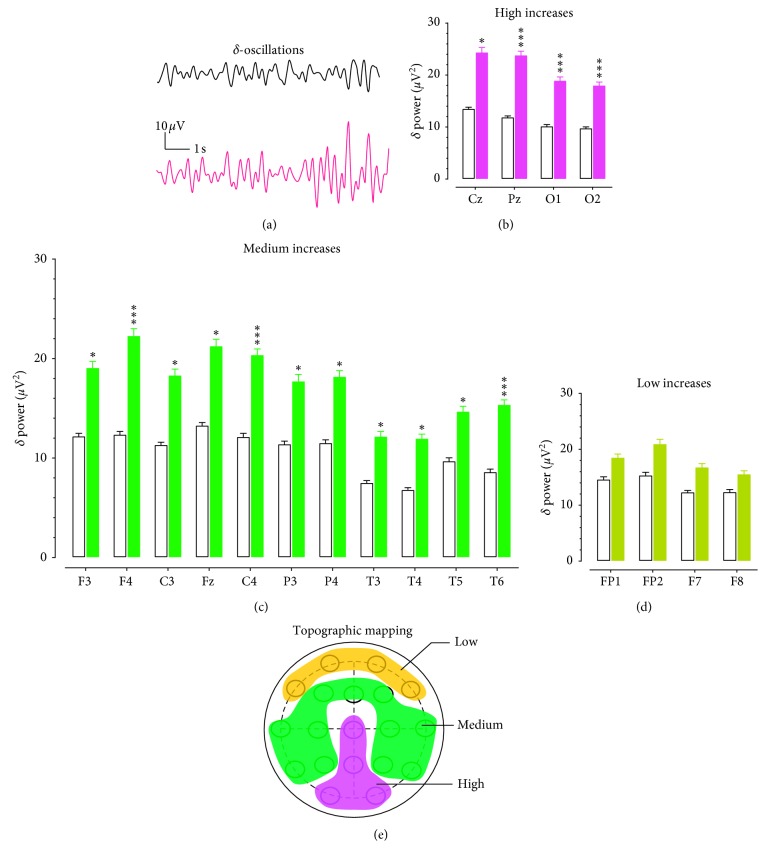
Topographic changes of *δ* powers in patients with opioid use disorder. (a) EEG traces of *δ* oscillations (1–4 Hz) representing 10 s activity from a healthy control (Top) and a patient with opioid use disorder. Horizontal scale bar, 1 s; vertical scale bar, 10 *μ*V. (b) The *δ* powers were increased by >76% in the Cz, Pz, O1, and O2 electrodes. Open columns denote healthy controls and solid red columns indicate the opioid group. ^*∗*^*P* < 0.05, ^*∗∗∗*^*P* < 0.001 vs. healthy controls determined by repeated measures ANOVA followed by *post hoc* Scheffe test. (c) The *δ* powers were increased by 51–75% in F3, F4, C3, Fz, C4, P3, P4, T3, T4, T5, and T6. Open columns denote the healthy control group, and solid green columns indicate the opioid group. ^*∗*^*P* < 0.05, ^*∗∗∗*^*P* < 0.001 vs. healthy control determined by repeated measures ANOVA followed by *post hoc* Scheffe test. (d) The *δ* powers were increased by 11–50% in FP1, FP2, F7, and F8. Open columns denote the healthy control group, and solid brown columns indicate the opioid group. NS, *P* > 0.05 vs. healthy control determined by repeated measures ANOVA. (e) Topographic correlation between increased *θ* oscillation (1–4 Hz) and electrodes on cortical scalps. The red area denotes high *δ* powers, the green for the medium *δ* powers, and the brown for the low *δ* powers.

**Figure 2:**
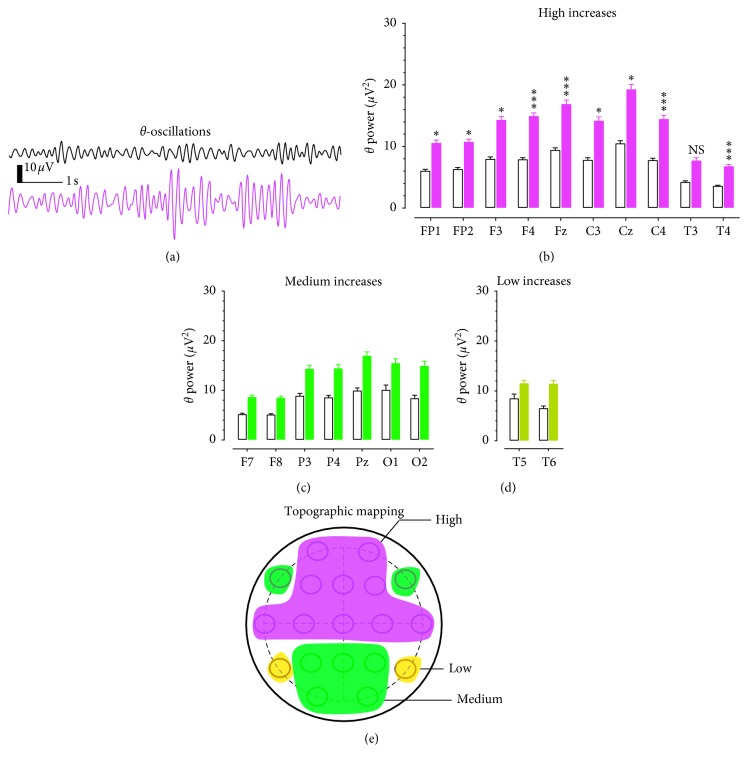
Topographic changes of *θ* powers in patients with opioid use disorder. (a) Examples of EEG traces representing 10 s *θ* oscillations from a healthy control (top) and a patient with opioid use disorder. Horizontal scale bar, 1 s; vertical scale bar, 10 *μ*V. (b) The *θ* powers was increased by >76%, including FP1, FP2, F3, F4, Fz, C3, Cz, C4, T3, and T4. Open columns denote healthy controls, and solid red columns indicate the opioid group. NS, *P* > 0.05, ^*∗*^*P* < 0.05, ^*∗∗∗*^*P* < 0.001 vs. healthy controls determined by repeated measures ANOVA followed by *post hoc* Scheffe test. (c) The *θ* powers were increased by 51–75% in F7, F8, P3, P4, Pz, O1, and O2. Open columns denote the healthy control group, and solid green columns indicate the opioid group. NS, *P* > 0.05 vs. healthy control determined by repeated measures ANOVA. (d) The *θ* powers were increased by 11–50% in T5 and T6. Open columns denote the healthy control group, and solid brown columns indicate a low change in the opioid group. NS, *P* > 0.05 vs. healthy control determined by repeated measures ANOVA. (e) Topographic correlation between increased *θ* oscillation (4–8 Hz) and electrodes on cortical scalps. The red area denotes for high *δ* powers, the green for medium *δ* powers, and the brown for low *δ* powers.

**Figure 3:**
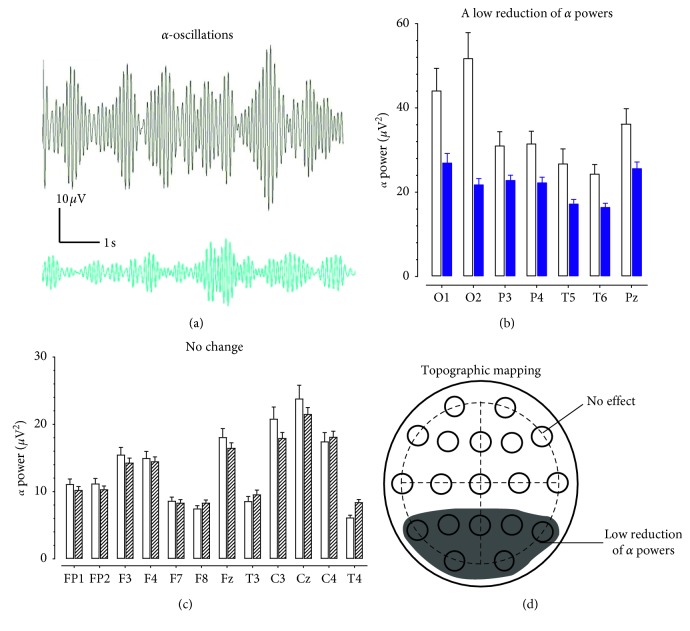
Topographic changes of *α* powers in patients with opioid use disorder. (a) Examples of EEG traces representing 10 s *α* oscillations from a healthy control (top) and a patient with opioid use disorder. Horizontal scale bar, 1 s; vertical scale bar, 10 *μ*V. (b) The *α* powers was reduced by 11–50% in O1, O2, P3, P4, T5, T6, and Pz. Open columns denote for the healthy control group and solid blue columns for a reduction of *α* oscillations in the opioid group. NS, *P* > 0.05 vs. healthy controls determined by repeated measures ANOVA. (c) There was no change of *α* powers in FP1, FP2, F3, F4, F7, F8, Fz, T3, C3, Cz, C4, or T4. Open columns denote for the healthy control group, and zebra-striping columns for the opioid group. NS, *P* > 0.05 vs. healthy control determined by repeated measures ANOVA. (d) Topographic correlation between reduced *α* oscillation (8–12 Hz) and electrodes on the scalp. The dark area denotes for a low reduction of *α* powers.

**Figure 4:**
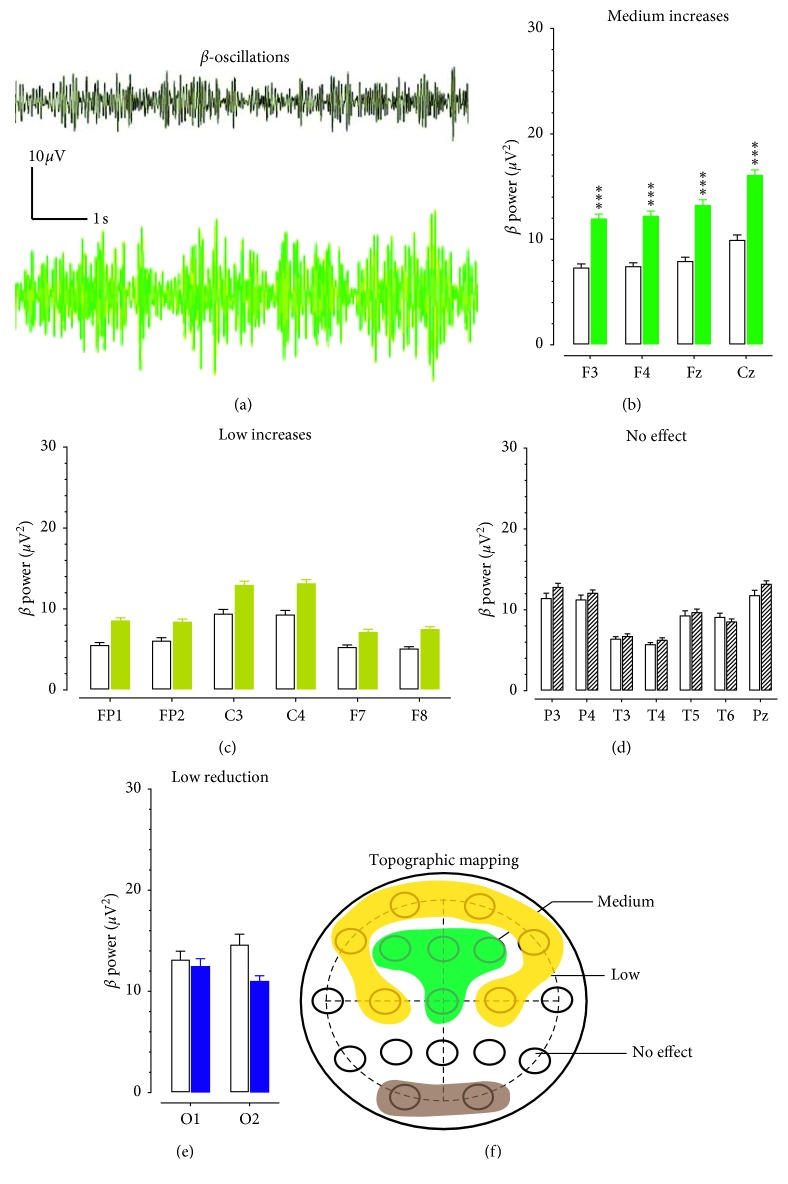
Topographic changes of *β* powers in patients with opioid use disorder. (a) Examples of EEG traces representing 10 s *β* oscillations from a healthy control (top) and a patient with opioid use disorder. Horizontal scale bar, 1 s; vertical scale bar, 10 *μ*V. (b) The increase of *β* powers was 51–75%, including F3, F4, Fz, and Cz. Open columns denote for the healthy control group and solid green columns for the opioid group. NS, *P* > 0.05. ^*∗∗∗*^*P* < 0.001 vs. healthy controls determined by repeated measures ANOVA followed by *post hoc* Scheffe test. (c) The increase of *β* powers was 11–50% in FP1, FP2, C3, C4, F7, and F8. Open columns denote for the healthy control group and solid brown columns for a low change in the opioid group. NS, *P* > 0.05 vs. healthy control determined by repeated measures ANOVA. (d) The increase of *β* powers was <10% in P3, P4, T3, T4, T5, T6, and Pz. Open columns indicate the healthy control group, and zebra-striping columns denote for “no change” in the opioid group. NS, *P* > 0.05 vs. healthy control determined by repeated measures ANOVA. (e) A tendency of *β* powers reduction was found in O1 and O2. Open columns denote for the healthy control group, and solid blue columns for a reduction of *β* oscillations in the opioid group. NS, *P* > 0.05 vs. healthy control determined by repeated measures ANOVA. (f) Topographic correlation between *β* oscillation (12–30 Hz) and electrodes on the scalp. The green area denotes for the medium increase of *β* powers, the brown for the low increases of *β* powers, and the dark for the low reduction of *β* powers.

**Figure 5:**
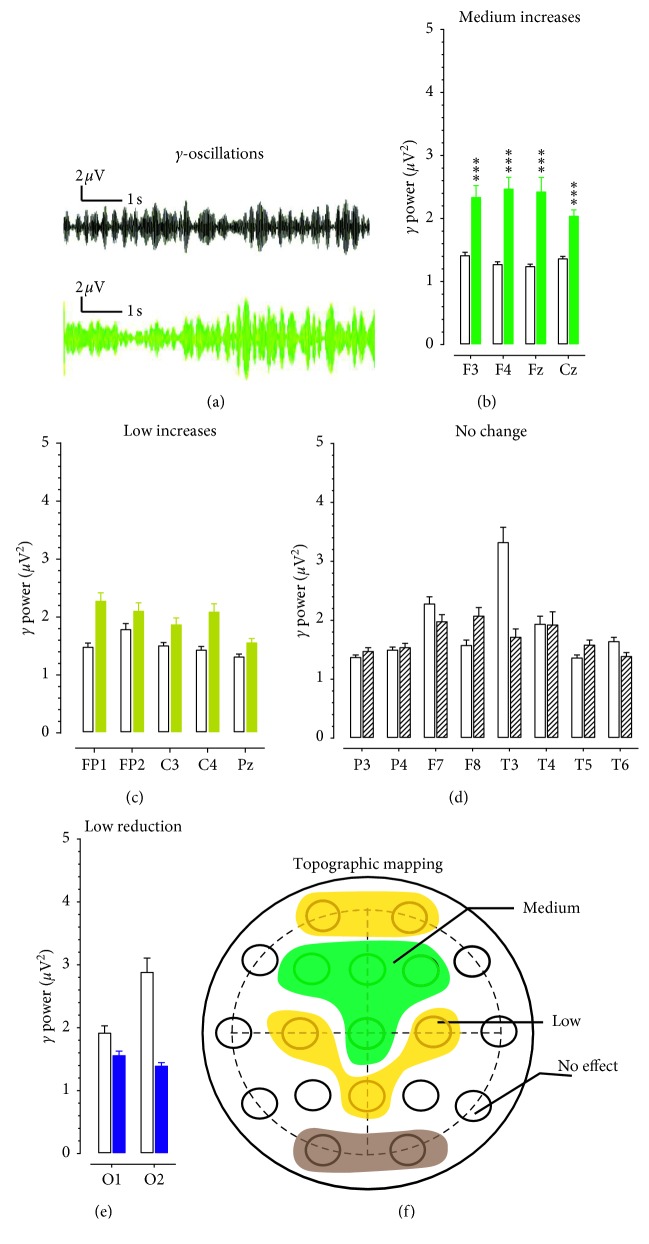
Topographic changes of *γ* powers in patients with opioid use disorder. (a) Examples of EEG traces representing 10 s *γ* oscillations from a healthy control (top) and a patient with opioid use disorder. Horizontal scale bar, 1 s; vertical scale bar, 2 *μ*V. (b) The increase of *γ* powers was 51–75%, including F3, F4, Fz, and Cz. Open columns denote for the healthy control group, and solid green columns for the opioid group. NS, *P* > 0.05. ^*∗∗∗*^*P* < 0.001 vs. healthy controls determined by repeated measures ANOVA followed by *post hoc* Scheffe test. (c) The increase of *β* powers was 11–50% in FP1, FP2, C3, C4, F7, and F8. Open columns denote for the healthy control group, and solid brown columns for a low change in the opioid group. NS, *P* > 0.05 vs. healthy control determined by repeated measures ANOVA. (d) The increase of *β* powers was <10% in P3, P4, T3, T4, T5, T6, and Pz. Open columns denote for the healthy control group, and zebra-striping columns for “no change” in the opioid group. NS, *P* > 0.05 vs. healthy control determined by repeated measures ANOVA. (e) A tendency of reduction in *β* powers was found in O1 and O2. Open columns denote for the healthy control group, and solid blue columns for a reduction of *γ* oscillations in the opioid group. NS, *P* > 0.05 vs. healthy control determined by repeated measures ANOVA. (f) Topographic correlation between *γ* oscillation (30–50 Hz) and electrodes on the scalp. The green areas denote for a medium increase of *γ* powers, the brown for the low *γ* powers, and the dark area for a low reduction of *γ* powers.

**Figure 6:**
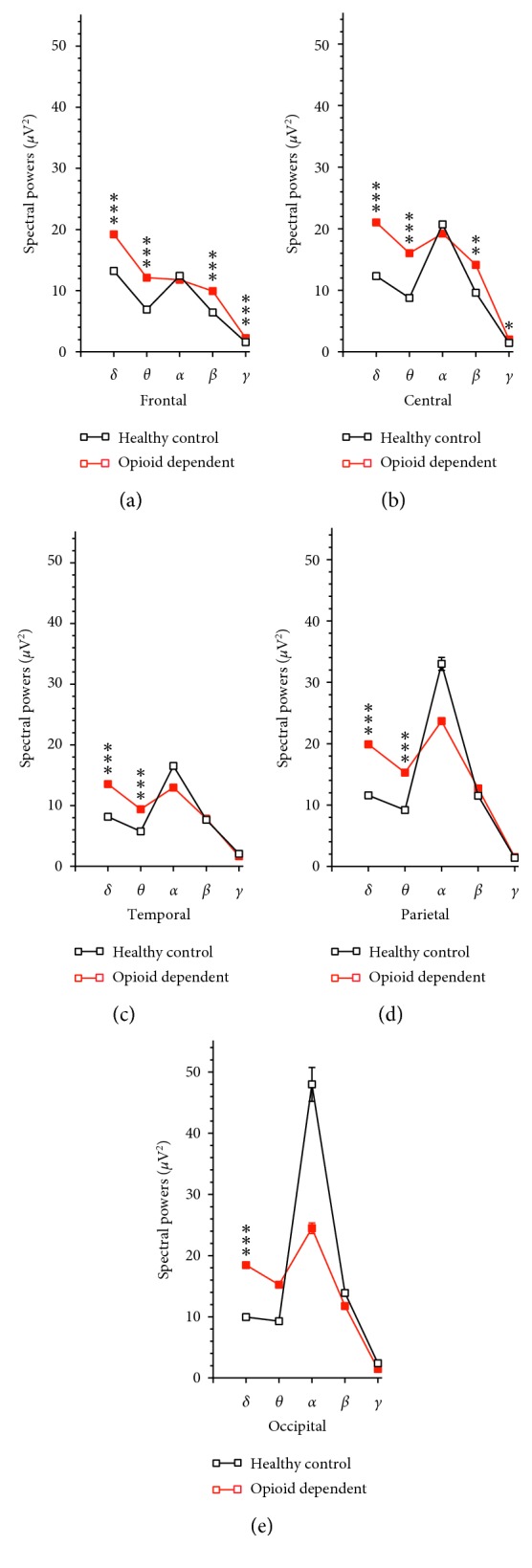
Comparative analysis of the 5 areas. (a) Compared to the respective frequency bands in health controls, there were increases in powers of *δ*, *θ*, *β*, and *γ* oscillations, but not *α* oscillations of patients with opioid use disorder. ^*∗∗∗*^*P* < 0.001 vs. health controls determined by unpaired Student's *t*-test. (b) Compared to the respective frequency bands in health controls, there were increases in powers of *δ* and *θ* oscillations, but not *α*, *β*, or *γ* oscillations of patients with opioid use disorder. ^*∗∗∗*^*P* < 0.001 vs. health controls determined by unpaired Student's *t*-test. (c) Compared to the respective frequency bands in health controls, there were increases in powers of *δ*, *θ*, *β,* and *γ* oscillations, but not *α* oscillations of patients with opioid use disorder. ^*∗∗∗*^*P* < 0.001 vs. health controls determined by unpaired Student's *t*-test. (d) Compared to the respective frequency bands in health controls, there were increases in powers of *δ* and *θ* oscillations, but not *α*, *β*, or *γ* oscillations of patients with opioid use disorder. ^*∗∗∗*^*P* < 0.001 vs. health controls determined by unpaired Student's *t*-test. (e) Compared to the respective frequency bands in health controls, there were increases in powers of *δ* oscillations, but not *θ*, *α*, *β*, or *γ* oscillations of patients with opioid use disorder. ^*∗∗∗*^*P* < 0.001 vs. health controls determined by unpaired Student's *t*-test.

**Figure 7:**
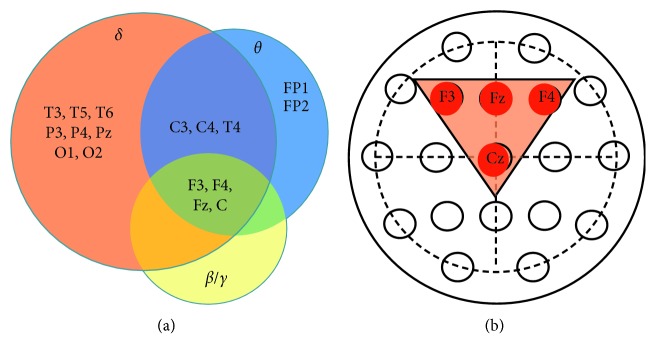
Venn diagram analysis to identify specificity of electrodes in which spectral powers were altered in patients with opioid use disorder. (a) The brown circle indicates *δ*-specific electrodes, the blue circle for *θ*-specific electrodes, and the yellow for *β*/*γ*-specific electrodes. (b) Topographic location of electrodes showing increases in *δ*, *θ*, *β,* and *γ* powers.

EEG data were downloaded from the database as described previously. Raw data were edited using the editing tool within the NeuroGuide software to remove physical artifacts (including eye movement, jaw movement, and gross movement). Test-retest reliability was used to govern EEG data quality after removal of the aforementioned artifacts. Theoretically, about 95% of the maximum change in EEG rhythms is reached within minutes of recording time, and longer EEG time is not necessary to capture the bulk of the variability in EEG spectra. To estimate reliability, EEG spectral powers at a selected segment or epoch (i.e., 20 sec, 40 sec, or 60 sec) are compared to those obtained from the whole segment (e.g., 10 min epoch). Reliability (*R*) is equal to 0 if there is no regression of spectral powers between two epochs, or 1 if the same results are achieved from two epochs. It has been shown that 20 sec epochs are typically to have 0.8 reliable, 40-sec epochs about 0.9 reliable, and 60-sec epochs at 0.95 reliable [29]. In the present study, we decided to use 60-sec epoch with reliable levels at 0.90 or greater. Nevertheless, a 60-sec epoch randomly selected from the artifact-free EEG graph (*R* ≥ 0.9) was subjected to EEG spectral power analysis using a fast Fourier transform (FFT), by which graphic images were mathematically transformed into voltage powers showing on *y*-axis (*μ*V^2^/Hz) plotted against spectral frequencies on *x*-axis (1–50 Hz; *δ*, 1–4 Hz; *θ*, 4–8 Hz; *α*, 8–12 Hz; *β*, 12–30 Hz; and γ, 30–50 Hz). The transformed numbers including both spectral powers (*μ*V^2^/Hz) and frequencies (Hz) were then copy-pasted to a Microsoft Excel sheet for further data calculation. Powers of *δ*, *θ*, *α*, *β,* or γ oscillations were individually sorted according to electrodes and averaged (mean ± SEM). The relationships between 5 spectral powers and 19 electrodes were determined in three distinct ways. First, the normal distribution of spectral powers (*δ*, *θ*, *α*, *β*, and γ) across different parts of the cortex was characterized. Specifically in healthy controls, data obtained from different areas were compared, including the frontal versus rear components, the left versus right components, and further dissected into the frontal, temporal, central, parietal, and occipital components. With such groundwork, we revealed differences in power levels between brain areas or lobes. Results of this analysis are presented in Tables 2–4. Next, spectral powers of *δ*, *θ*, *α*, *β,* or γ oscillations at individual electrodes in patients with opioid use disorder were compared with those of healthy controls, across all electrode sites (frontal: FP1, FP2, F3, F4, Fz, F7, and F8; central: C3, Cz, and C4; temporal: T3, T4, T5, and T6; and parietal-occipital: P3, Pz, P4, O1, and O2). To reveal topographic distribution of spectral powers in the brain, power levels were arbitrarily categorized into four groups as follows: high >76%; medium 51–75%; low 11–50%; no change <10%. After initial analysis, we found that for most cases the spectral powers of counterpart electrodes at two hemispheres were almost identical, and thus the counterpart electrodes were always grouped together. There were only a few cases that the power levels were different between two hemispheres; if it occurred, the counterpart electrodes were categorized into the low-level change group. Results of the analysis are presented in Figures 1–5. The third analysis was to reveal what EEG electrodes could have more specific changes than others. To fulfill this aim, data were analyzed from several aspects, including analyses of spectral powers, brain lobes, or EEG electrodes. Furthermore, Venn diagram analysis was used to determine specificity of electrodes that could detect changes in spectral powers, including all 5 spectra (i.e., *δ*, *θ*, *α*, *β,* and γ oscillations). Results of this analysis are presented in Table 5 and Figures 6 and 7.

### 2.4. Statistical Analysis

All data are expressed as mean ± SEM and have been evaluated with repeated measures ANOVA between subjects (e.g., frontal vs. rear areas and patients vs healthy controls) followed by *post hoc* Scheffe test using StatView software 5.0 (SAS Institute Inc., Cary, NC, USA). Unpaired Student's *t*-test was also utilized to determine statistical differences if appropriated. Significance was set at 0.05.

## 3. Results

### 3.1. A Normal Distribution of Spectral Powers across the Scalp in Healthy Controls

First, we analyzed 16 healthy controls to determine whether spectral powers of *δ*, *θ*, *α*, *β,* or γ oscillations were different between the frontal (FP1, FP2, F3, F4, F7, F8, and Fz) versus rear scalps (O1, O2, P3, P4, T5, T6, and Pz). As illustrated in Table 2, *δ* powers were significantly higher in the frontal sites compared to the rear sites (*F*_(1,222)_ = 14.188, *P*=0.0002). There was no significant difference in *θ* powers between the two areas (*F*_(1,222)_ = 0.522, *P*=0.4708). As the frequencies increased to *α* or *β* oscillation, spectral powers were significantly lowered in the frontal sites relative to the rear sites (*α*, *F*_(1,222)_ = 14.027, *P*=0.0002; *β*, *F*_(1,222)_ = 22.143, *P* < 0.0001). For the γ oscillations, the powers at the frontal sites were almost the same as the rear sites, showing no statistical difference (*F*_(1,222)_ = 0.522, *P*=0.4708).

Next, spectral powers on the left hemisphere (FP1, F3, F7, C3, T3, F3, T5, and O1) were compared with those of the right (FP2, F4, F8, C4, T4, F4, T6, and O2). Data analysis revealed no difference in spectral powers between the two hemispheres (Table 3).

Lastly, electrodes were regrouped into the frontal (FP1, FP2, F3, F4, F7, F8, and Fz), central (C3, C4, and Cz), temporal (T3, T4, T5, and T6), parietal (P3, P4, and Pz), and occipital (O1 and O2) areas. Spectral powers were significantly different between those areas (Table 4). Further data analysis revealed that *α* and *β* powers were low in the frontal areas and markedly increased in a range of 100%–300% in the occipitals. In contrast, changes of *δ*, *θ*, and γ powers were limited, with less than 50% difference between areas.

### 3.2. Changes in Powers of Patients with Opioid Use Disorders

#### 3.2.1. Changes in *δ* Powers

Figure 1(a) displays samples of 10 s *δ*-oscillations obtained from a Cz electrode placed in the midline center on the scalp. It appears that the *δ* powers were increased in a patient with opioid use disorder (Figure 1(a) bottom) as compared with an age- and sex-matched healthy subject (Figure 1(a) top). Changes of *δ* powers across 19 EEG electrodes were arbitrarily classified into 4 levels: high (>76%), medium (51–75%), low (11–50%), and no change (<10%).  Figure 1(b) shows 4 electrodes having more than >76% increases in *δ* powers. The results of two-factor ANOVA indicated a significant main effect for opioid use (*F*_(1,30)_ = 7.874, *P*=0.0087) and a significant main effect for electrode locations (*F*_(18,540)_ = 10.475, *P* < 0.00001). Specifically, all were located in the midline centers and occipitals, including Cz, Pz, O1, and O2. In the healthy controls, *δ* powers (*μ*V^2^; *N* = 16) were 13.5 ± 0.3, 11.7 ± 0.3, 10.2 ± 0.3, and 9.8 ± 0.3, respectively. The *δ* powers in those electrodes of opioid patients were increased by 80%, 101%, 86%, and 84%, respectively.

Figure 1(c) shows a medium increase (51–75%) of *δ* powers in the frontal (F3 and F4), central (C3, Fz and C4), parietal (P3 and P4), and temporal areas (T3, T4, T5, and T6). The increase was significant (*F*_(1,30)_ = 8.726, *P*=0.0061).

Figure 1(d) shows an 11–50% increase of *δ* powers in the FP1, FP2, F7, and F8 electrodes. However, the increased *δ* powers were not statistically different from those of the healthy controls (*F*_(1,30)_ = 2.115, *P*=0.156).  Figure 1(e) displays topographical mapping of distribution of *δ* powers categorized with high, medium, and low levels. The red area denotes a high-activity increase of *δ* powers, the green for the medium-activity increase, and the brown for the low-activity increase of *δ* powers.

#### 3.2.2. Changes in *θ* Powers

Figure 2(a) displays samples of 10 s *θ* oscillations obtained from the FP1 electrode in a patient with opioid use disorder (Figure 2(a) bottom) as compared to a healthy control (Figure 2(b) top). Figure 2(b) shows the group of electrodes recorded with high *θ* power changes (>76% vs. healthy control). They were located in the frontal (FP1, FP2, F3, and F4), middle-central (Fz, C3, Cz, and C4), and temporal areas (T3 and T4). Two-way repeated measures ANOVA revealed a significant main effect for opioid patients (*F*_(1,30)_ = 4.792, *P*=0.037) and a significant main effect for the 19-electrode placements (*F*_(18,540)_ = 13.135, *P* < 0.0001). Except for the T3 electrode, *post hoc* Scheffe test revealed the increase was significantly different from the respective electrodes of the healthy controls.

Overall, a medium-activity increase (51–75%; Figure 2(c)) of *θ* powers was found in 7 electrodes (Figure 3(c); F7, F8, P3, P4, Pz, O1, and O2). Statistical analysis revealed that *θ* changes at those 7 electrodes were not different from the control group (*P* > 0.05). A low increase (11–50%; Figure 2(d)) of *θ* powers was found in the T5 and T6 electrodes, and this effect was not significant (*P* > 0.05). Topographical changes of *θ* powers on the scalp are shown in Figure 2(e). The red area denotes a high increase of *θ* powers, the green for the medium increase, and the brown for the low increase of *θ* powers.

#### 3.2.3. Changes in *α* Powers

Figure 3(a) displays samples of 10 s *α* oscillations obtained from the O1 electrode in a patient in contrast to the healthy controls. There was a low reduction (10–50%) of *α* oscillations in the O1, O2, T5, T6, P3, P4, and Pz electrodes (Figure 3(b)). However, the reduction was not statistically different from that obtained from the healthy control (*F*_(1,30)_ = 0.368, *P*=0.549).

Changes in the remaining electrodes were less than 10%, including FP1, FP2, F3, F4, F7, F8, Fz, T3, C3, Cz, C4, and T4 (Figure 3(c)). The dark area in Figure 3(d) represents topographical reduction of the *α* powers in the temporal, parietal, and occipital areas.

#### 3.2.4. Changes in *β* Powers

Figure 4(a) displays samples of 10 s *β* oscillations obtained from F3 electrodes. The *β-*amplitude was increased in a patient with opioid use disorder (Figure 5(a) bottom) as compared to a healthy control (Figure 4(a) top). However, no activity exceeding 76% was found in any electrode.  Figure 4(b) shows the group of electrodes recorded with medium *δ* power changes (51–75%). Results of two-way repeated measures ANOVA show a significant main effect for opioid patients (*F*_(1,30)_ = 10.359, *P*=0.031) and a significant main effect for the 19-lead electrode placements (*F*_(18,540)_ = 12.167, *P* < 0.0001). A low increase (11–50%) was found in the FP1, FP2, C3, C4, F7, and F8 electrodes (Figure 4(c)). The change had no statistically significant difference from the respective electrodes obtained from the healthy controls (*F*_(1,30)_ = 2.928, *P*=0.097). Changes in the P3, P4, T3, T4, T5, T6, and Pz electrodes (Figure 4(d)) were less than 10% and not different from the controls (*F*_(1,30)_ = 0.091, *P*=0.766). Lastly, *β* powers obtained from O1 and O2 electrodes were evaluated, finding a tendency of a low reduction in the occipitals (Figure 4(e)). However, the reduction was not statistically different from the healthy controls (*F*_(1,30)_ = 0.238, *P*=0.629).  Figure 4(f) displays *β* power levels topographically distributed in the scalp. The green area denotes a medium increase of *β* powers, the brown for the low increase, and the dark represents the low reduction of *β* powers.

#### 3.2.5. Changes in γ Powers

Figure 5(a) displays samples of 10 s γ-oscillations obtained from F3 electrodes, showing increases of γ-amplitudes in an opioid patient (bottom) in contrast to a healthy control (top).  Figure 5(b) shows the group of electrodes recorded with a medium *γ* power increase, including F3, F4, Fz, and Cz electrodes. The results of two-factor ANOVA indicated a significant main effect for opioid use (*F*_(1,30)_ = 54.084, *P* < 0.001) and a significant main effect for electrode locations (*F*_(18,540)_ = 15.268, *P* < 0.00001).  Figure 5(c) shows a low increase of *γ* powers in the FP1, FP2, C3, C4, and Pz electrodes. However, the effect was not significant (*F*_(1,30)_ = 1.287, *P*=0.266).  Figure 5(d) shows a group of electrodes had no change in *γ* powers, including P3, P4, F8, T3, T4, T5, and T6 (*F*_(1,30)_ = 0.150, *P*=0.702). Note that, although T3 powers were reduced by 48%, it would be classified into “no effect” since T4 had less than 1% change compared with the electrode in the counterpart area. There was a tendency that O1 and O2 electrodes had a reduction in *γ* powers (Figure 5(e)). However, this effect was not significant (*F*_(1,30)_ = 1.932, *P*=0.175).  Figure 5(f) displays a topographical response of *γ* powers in opioid patients. The green area indicates medium increases of *γ* powers, the brown for the low increases, and the dark represents the low reduction of *γ* powers as measured at the scalp.

### 3.3. Identifying Specificity of Electrodes That Could Be Used to Detect Changes in EEG Powers of Patients with Opioid Use Disorders

#### 3.3.1. Aberrant Distribution of Spectral Powers Across the Scalp in Patients with Opioid Use Disorder

In addition to the individual analysis, electrodes were grouped as the frontal (FP1, FP2, F3, F4, F7, F8, and Fz), temporal (T3, T4, T5, and T6), central (C3, C4, and Cz), parietal (P3, P4, and Pz), and occipital electrodes (O1 and O2).  Figure 6 shows the mean powers in these areas of the patients compared with healthy controls. The *δ* powers were increased throughout all 5 areas. The *θ* powers were increased in 4 areas except for the occipitals. Although there was a reduction of *α* powers in sensorimotor regions (i.e., temporal, parietal, and occipital areas), this effect was not significant. The *β* and *γ* powers were increased mainly in the frontal and central areas, but not in temporal or parietal areas, and a nonsignificant reduction in occipitals.

#### 3.3.2. Venn Diagram Analysis to Identify Electrodes That Could Detect Changes in Powers of Spectra as Many as Possible, including *δ*, *θ*, *β,* and *γ* Oscillations

Individual electrodes which had significantly increased powers (based on results from Figures 1–6) are sorted into *δ*, *θ*, or *β*/*γ* groups with a Venn diagram. As shown in Figure 7(a), 15 out of 19 electrodes are found in the *δ* group. In contrast, only 9 electrodes are found in the *θ* group. C3, C4, T4, F3, F4, Fz, and Cz in the *δ* group are overlapped with *θ* groups. Both *β* and *γ* groups have F3, F4, Fz, and Cz. Overall, F3, F4, Fz, and Cz are overlapped by *δ*, *θ*, *β*/*γ* groups. Topographically, those 4 electrodes were next each other on the scalp (Figure 7(b)).

## 4. Discussion

The current study yielded several major findings. First, there was topographical distribution of *δ*, *θ*, *α*, *β,* and γ powers across the scalp of healthy controls in a closed-eye resting state. The *δ* powers were high in the frontal areas, while *α* and *β* powers were dominant in the rear areas, particularly in the occipitals. However, the distribution pattern for *θ* and *γ* powers was not apparent. Nevertheless, the data support the notion that spectral powers are characteristically distributed across the cortex, which can be considered as fundamental values in estimating mental health. Second, we found that changes in spectral powers were characteristic of patients with opioid use disorder, consistent with previous opioid research reports [26, 30]. A majority of 19 electrodes could detect an increase of *δ* but not *θ*, *α*, *β*, or *γ* powers. Interestingly, all except for the *α* component increased at F3, F4, Fz, and Cz. This suggests that these four electrodes may be more useful than others in assessing opioid use disorder. Third, we observed that *α* powers were reduced in the sensorimotor areas, particularly occipital O1 and O2 electrodes. Although the reduction was not statistically significant, it suggests that *α* oscillations in response to opioid use was different from other oscillations.

### 4.1. Topographic Analysis of Spectral Powers across the Scalp in Healthy Controls

In this study, EEG microstates exhibited two apparent patterns. First, EEG activity obtained from a given electrode was very similar to nearby or surrounding electrodes. As a result, changes in spectral powers appear gradual rather than sudden. For instance, Cz is located in the midline center surrounded by C3, Fz, C4, and Pz. EEG activity on Cz was more or less similar to C3, Fz, C4, or Pz. This pattern could be applied to almost any other electrode, and thus changes in spectral powers of several nearby electrodes could be grouped for the data analysis. Second, the corresponding or counterpart electrodes located on two hemispheric sides (e.g., F7 vs. F8 and T5 vs. T6) had almost the same power levels and the same response to opioid use. One explanation for the similarity in power may be ascribed to the fact that there are the same or similar neural anatomies or microcircuits between two hemispheres, and thus the distribution of power levels appears to be symmetric as measured at the scalp. This also suggests that the apparatus used in the study had high quality in collecting EEG data.

However, there was a regional difference in power between frontal and rear areas, and between lobes (frontal, temporal, central, parietal, and occipital). The *δ* oscillations appear higher in the frontal area while *α* and *β* are higher in the occipital area, consistent with previous observations in healthy controls [31–33]. The difference in spectral powers can be ascribed to the cortical microcircuits beneath electrodes. It has been observed that the dendritic fields of pyramidal neurons in the frontal cortex are several times greater than those in the occipital cortex [34]. In addition, deep brain projections to cortical layers may be different between cortical lobes. For instance, thalamic neurons provide greater inputs to the visual cortex while the hippocampal, ventral tegmental area (VTA), and many other nuclei predominately innervate the frontal cortex [35–37]. Thus, differences in the microcircuits would be the cause of differential powers of spectra received on the EEG electrodes, consistent with previous reports [38]. Taken together, levels of spectral powers may be characteristically distributed across scalps.

### 4.2. Increases in *δ*, *θ*, *β* and γ Powers, but Not *α* Power, of Patients with Opioid Use Disorder

We found that, between 19 electrodes, there were 15 electrodes showing significant increases in *δ* oscillations, or 74% of total electrodes. In contrast, there were only 9 (47%), 4 (21%), and 4 electrodes (21%) showing significant increases in *θ*, *β*, and *γ* powers, respectively. The findings are in line with some of the previous reports [39], but not others [30], demonstrating that *δ* oscillations are the major frequency band altered in patients with opioid use disorder. This suggests that *δ* oscillations are more reliable than other frequencies in estimating opioid use disorder. However, an individual electrode analysis could underestimate the importance of other powers (i.e., *θ*, *α*, *β*, or *γ*). For this reason, we also conducted group-oriented Venn diagram analysis highlighting significance in the frontal, central, temporal, parietal, and occipital areas. Results of our data analysis based on several approaches prompted us to suggest that changes of spectral powers in response to opioid use can be grouped into three subclasses. As illustrated in Table 5, the *δ* and *θ* powers could be grouped together as class 1, showing significant increases in almost all areas, suggesting that opioids likely modulate *δ* and *θ* activity across the cortex. The *β* and *γ* powers grouped as class 2 were mainly found in the frontal and central areas, implicating involvement of emotional control, not sensorimotor activity located in the temporal or parietal regions. Given that dopaminergic projections from the VTA to cortical areas mainly target the frontal regions [40], it would be of interest to explore whether dopaminergic activity altered by opioids is involved in changes of the *β* and *γ* powers. The *α* powers as class 3 could be exceptional and different from either class 1 or 2, showing no increase on any region measured. Instead, there was a reduction of powers in the sensorimotor areas, such as temporal, parietal, and occipital regions (Figure 3 and Table 5). An advantage of such a categorization is that it allows the spectral powers to associate with specific brain areas. However, additional verification is needed to determine whether functional activity relevant to areas is indeed modulated or impaired in association with changes in spectral activity, which could be explored with event-related potential (ERP) measurements in patients with opioid abuse [26].

### 4.3. Possible Mechanisms Responsible for Increases or Decreases in Spectral Powers

The increases in powers can be interpreted as increasing synchronized activity of pyramidal neurons in the cortical microcircuits as measured by nearby electrodes, consistent with EEG theory [41]. This suggests that the inhibitory and excitatory balances in the microcircuits of neuronal activity have been modulated in the opioid patients. Mechanisms are not fully understood, but changes in neuroplasticity could be one of the causes that contribute to the modulation. Functional neuroplasticity refers to the enhancement of synaptic strength, involving IPSPs or EPSPs or both (see reviews [42]). While there is no direct effect of opioids on pyramidal neurons, the increasing synchronized effect is ascribed mainly to synaptic inputs from either GABAergic interneurons or deep brain afferents. Electrophysiological studies revealed that signal transduction pathways of GABAergic interneurons are impaired, and the spontaneous IPSPs are significantly increased in brain slices of chronic opioid rodents reported in some studies [43–45], but not others [46]. Thus, increasing EEG synchronization may be due to increasing GABAergic inputs to the pyramidal neurons. Consistent with the hypothesis, it was found that opioids no longer cause a reduction in IPSPs of brain slices obtained from chronic opioid-treated animals, but instead potentiate the IPSPs [44, 47, 48]. GABAergic activity is found to be increased in the cortical microcircuits, specifically the medial prefrontal cortex [49].

Glutamatergic pyramidal neurons in the cortex are the major carriers in which plasticity takes place [50]; also see reviews by [51]. Consistent with this, glutamate levels were increased in the brain of patients with opioid use disorder [52, 53]. Signal transduction pathways, including NMDA–Ca^2+^–NO–cGMP pathway, AC–cAMP–CREB pathway, and MAPK pathways are activated, which are critical for plasticity changes [54]. Involvement of glutamatergic neuroplasticity was also supported with observation in animal studies. For instance, the noncompetitive NMDA receptor blocker, MK-801 antagonized the changes of EEG spectral powers [55], and EPSP amplitudes of glutamatergic neurons synapsing to pyramidal neurons in the cortex were increased in rodents with chronic opioids [56]. In addition, chronic use of opioids sensitizes the expression of D_1_ receptors on glutamatergic neurons in the basolateral amygdala that project to the cortex [56].

In addition to increases, we also observed reduction in spectral powers, mainly *α* oscillations in the sensorimotor areas measured with O1 and O2 electrodes. It is suggested that a reduction in power is indicative of desynchronization of neuronal activities associated with wakefulness, alertness, and intensive activity of the perceptional process in response to a task [57, 58]; also see an excellent review [59]. In contrast, increases in power may be attributed to synchronized IPSPs as patients become drowsy during cortical idling or active inhibition [60]. Increases in power are also associated with synchronized EPSPs for hallucinations [61], and abuse of psychostimulants [62, 63]. Despite this, the exact mechanism underlying relationship between changes in spectral powers and mental health is still not understood.

## 5. Conclusions

Consistent with previous reports [23, 26, 39, 64], we demonstrate that changes in spectral rhythms took place in some, but not all 19 electrodes of patients with opioid use disorder. Major changes were found at F3, F4, Fz, and Cz. Considering that EEG rhythms are electrical signals reflecting activity of neural circuits consisting of glutamatergic, GABAergic, and many other types of neurons, the findings of the present study are limited by the study design focusing on opioid cases only, and unlikely applicable to polysubstance users. This is because the detrimental effect of opioids is ascribed to mainly GABAergic dysfunction [18, 19], other types of neurons contributed to changes in EEG rhythms in polysubstance use was not examined in the present study. We found that patients with methamphetamine or alcohol use disorder were shown different changes in EEG rhythms (unpublished observation), supporting this hypothesis. Furthermore, the present investigation included only 16 matched cases. Strict validation with large sample sizes is needed before being generalized to all opioid users. It is worthy to note that EEG data were recorded while subjects were asked to have eyes closed. It would be interesting in the future study to investigate event-related potentials (ERP) in patients with opioid use disorders [26, 65].

## Figures and Tables

**Table 1:** Breakdown medical information of patients with opioid use disorder.

Subject ID	Age (year)	Sex	Ethnicity^a^	Drug^b^	Years on drug	Other health issues
O2	33	M	W	O, H	14	Bipolar disorder, unspecified
O3	29	M	W	H	6	N/A
O6	31	F	W	H	5	N/A
O7	44	M	W	H	20	Hepatitis C
O8	22	F	W	H	4	Hepatitis C
O13	24	M	Hi	H	5	Hepatitis C
O15	51	M	W	O	4	Hypertension
O17	56	M	Hi	O	5	Intermittent asthma
O18	49	M	W	O	3	Type II diabetes; hypertension
O26	35	M	W	O, H	15	Psoriasis vulgaris
O28	45	F	W	H	3	Attention-deficit hyperactivity disorder
O30	40	M	W	O	12	N/A
O31	29	M	W	H	7	N/A
O33	25	F	W	H	4	N/A
O34	49	M	Hi	O	13	Gastroesophageal reflux disease; hypertension
O35	22	F	W	H	4	N/A

^a^W denotes White; Hi, Hispanic. ^b^M denotes morphine; H, heroin; O, oxycodone.

**Table 2:** Comparative analysis of power levels distributed on the scalp of healthy controls. Data were based on 16 healthy controls.

	Frontal scalp (*F*; *μ*V^2^)^b^	Rear scalp (*R*; *μ*V^2^)	*F/R* ratio	*P* value^c^
*δ* ^a^	13.2 ± 0.1	10.4 ± 0.1	1.3	*P* < 0.001
*θ*	6.9 ± 0.1	8.8 ± 0.1	0.8	*P* > 0.05
*α*	12.4 ± 0.1	35.2 ± 0.6	0.4	*P* < 0.001
*β*	6.4 ± 0.1	11.6 ± 0.1	0.6	*P* < 0.001
*γ*	1.6 ± 0.1	1.7 ± 0.1	0.9	*P* > 0.05

^a^ *δ*, 1–4 Hz; *θ*, 4–8 Hz; *α*, 8–12 Hz; *β*, 12–30 Hz; and *γ*, 30–50 Hz. ^b^Frontal (F), FP1, FP2, F3, F4, F7, F8, and Fz); Rear (R), O1, O2, P3, P4, T5, T6, and Pz). Note that the electrode located in the middle (T3, T4, C3, C4, and Cz) were excluded in the data analysis. ^c^Significance was determined by repeated measures ANOVA.

**Table 3:** Comparative analysis of power levels distributed on the scalp of healthy controls. Data were based on 16 healthy controls.

	Left scalp (*L*; *μ*V^2^)	Right scalp (*R*; *μ*V^2^)	*L/R* ratio	*P* value
*δ*	11.2 ± 0.1	11.1 ± 0.1	1.0	>0.05
*θ*	7.4 ± 0.1	6.8 ± 0.1	1.1	>0.05
*α*	20.9 ± 0.3	20.7 ± 0.3	1.0	>0.05
*β*	8.5 ± 0.1	8.6 ± 0.1	1.0	>0.05
*γ*	1.8 ± 0.1	1.8 ± 0.1	1.0	>0.05

Note that comparative analysis of power levels recorded in the left (FP1, F3, F7, C3, T3, F3, T5, and O1) versus right scalps (FP2, F4, F8, C4, T4, F4, T6, and O2). The electrodes at midline (Fz, Cz, and Pz) were excluded from the data analysis.

**Table 4:** Comparative analysis of power levels distributed on scalp of healthy controls. Data were based on 16 healthy controls.

	Frontal^a^	Central^b^	Temporal^c^	Parietal^d^	Occipital
*δ*	13.2 ± 0.1	12.3 ± 0.1	8.2 ± 0.1^a,b^	11.6 ± 0.1^c^	10.0 ± 0.1^c^
*θ*	6.9 ± 0.1	8.8 ± 0.1	5.8 ± 0.1^b^	9.2 ± 0.1^c^	9.3 ± 0.4^c^
*α*	12.4 ± 0.1	20.7 ± 0.6	16.5 ± 0.5	33.1 ± 1.1^a,b,c^	48.1 ± 2.8^a,b,c^
*β*	6.4 ± 0.1	9.6 ± 0.2^a^	7.7 ± 0.1	11.5 ± 0.2^a,c^	13.9 ± 0.5^a,b,c^
*γ*	1.6 ± 0.1	1.5 ± 0.1	2.1 ± 0.1^b^	1.4 ± 0.1^c^	2.4 ± 0.1^a,b,d^

Note that electrodes placed on scalp of the frontal (FP1, FP2, F3, F4, F7, F8, and Fz), temporal (T3, T4, T5, and T6), central (C3, C4, and Cz), parietal (P3, P4, and Pz) and occipital areas (O1 and O2). Significance were determined by repeated measures ANOVA followed by *post hoc* Scheffe test. ^a^*P* < 0.05 vs. Frontal. ^b^*P* < 0.05 vs. Temporal. ^c^*P* < 0.05 vs. Central. ^d^*P* < 0.05 vs. Parietal.

**Table 5:** Classification of EEG spectral activity on scalps of patients with opioid use disorders. The activity was categorized into three classes as follows:

		Frontal		Central		Temporal		Parietal		Occipital	
Class 1	*δ*	↑	✓	↑	✓	↑	✓	↑	✓	↑	✓
	*θ*	↑	✓	↑	✓	↑	✓	↑	✓	↑	✗

Class 2	*β*	↑	✓	↑	✓	↔	✗	↔	✗	↓	✗
	*γ*	↑	✓	↑	✓	↔	✗	↔	✗	↓	✗

Class 3	*α*	↔	✗	↔	✗	↓	✗	↓	✗	↓	✗

Note that frontal electrodes included FP1, FP2, F3, F4, F7, F8, and Fz; central electrodes included T3, T4, T5, and T6; temporal were C3, C4, and Cz; parietal were P3, P4, and Pz; and occipitals were O1 and O2. ↑✓, Increases in spectral powers with statistical significance. ↑✗, Increases in spectral powers but no statistical significance. ↔✗, Neither increases nor decreases in spectral powers. ↓✗, Decreases in spectral powers with statistical significance.

## Data Availability

The authors declare that data supporting the findings of this study are available within the article.
